# Data-Driven Control of Neuronal Networks with Population-Level Measurement

**DOI:** 10.21203/rs.3.rs-2600572/v1

**Published:** 2023-03-17

**Authors:** Minh Vu, Bharat Singhal, Shen Zeng, Jr-Shin Li

**Affiliations:** Department of Electrical and Systems Engineering, Washington University in St. Louis, MO, USA

## Abstract

Controlling complex networks of nonlinear neurons is an important problem pertinent to various applications in engineering and natural sciences. While in recent years the control of neural populations with comprehensive biophysical models or simplified models, e.g., phase models, has seen notable advances, learning appropriate controls directly from data without any model assumptions remains a challenging and less developed area of research. In this paper, we address this problem by leveraging the network’s local dynamics to iteratively learn an appropriate control without constructing a global model of the system. The proposed technique can effectively regulate synchrony in a neuronal network using only one input and one noisy population-level output measurement. We provide a theoretical analysis of our approach and illustrate its robustness to system variations and its generalizability to accommodate various physical constraints, such as charge-balanced inputs.

## 1. Introduction

Large populations of interacting neurons govern the majority of our physiological activities, from circadian and central nervous system rhythms [1, 2] to memory formation [3]. These populations often require precise firing patterns among units to operate effectively, for example, synchronization of neural activity in the gamma band for perceptual and cognitive functions [4, 5] and desynchronization for transition between perception and the motor response [6]. Disruptions in these patterns lead to pathological conditions such as Parkinson’s disease [7], epilepsy [8], and schizophrenia [9], where excessive synchronization of neuronal activities is detected.

**Figure 1.**
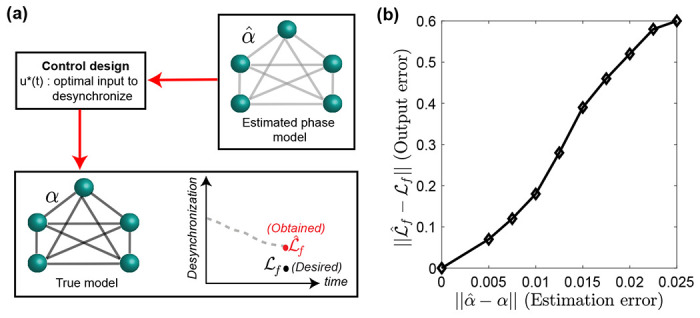
Panel (a) displays the error in the final desynchronization index induced by an optimal control design based on the estimated phase model. The symbol ${ℒ}_{f}$ denotes the desired desynchronized index, evaluated using the order parameter (see Methods), whereas ${\hat{ℒ}}_{f}$ is the desynchronized index obtained by the optimal input $u^{*}(t)$. In panel (b), we consider minimum-energy controls designed from true and the estimated phase model and evaluate the error in the final desynchronization index as the estimation error varies. The estimation error represents the incorrect estimate of the coupling strength, $\left\|\hat{\alpha }-\alpha \right\|$, and the minimum-energy control is designed by using optimal control theory (see Supplementary Materials for details).

Owing to these practical interests, several techniques to regulate the synchronization patterns in neural populations have been proposed. These techniques, although effective, rely on either state-space descriptions [10, 11] or reduced-order models such as phase models [12–20], both of which have their own set of limitations. The state-space control design, for example, suffers from (i) partial observability, due to the availability of a single measurement such as local field potential, which makes identifying the state-space model difficult [21], and (ii) the high dimensionality which hinders the control design, assuming that the true model can even be obtained. Phase models, on the other hand, facilitate tractable control design due to their low dimensionality but are only applicable to small-magnitude control inputs [1], and the precise input bounds are often unknown in practice [22]. Most importantly, inferring the true coupling dynamics is a non-trivial task [23, 24] where estimation errors could result in unsatisfactory control performance (Figure 1).

Given these limitations associated with model-based approaches and the recent unprecedented availability of measurement data, it is therefore natural to ask whether the desired control strategies can directly be learned from data. In this paper, we address this problem by designing control inputs that can synchronize or desynchronize a network of neurons within a pre-specified time frame. The advocated framework differs from the existing works [25–31] in that it does not require the availability of a rich training data set but instead leverages the system local dynamics to directly learn appropriate control with a small number of trails. We learn the control input online by iteratively perturbing the system and observing its response. We present two output measurement scenarios: (i) when voltages of individual neurons can be measured, and (ii) when only a single measurement in the form of the mean firing voltage is available. The effectiveness of the developed control framework is demonstrated on a network of Hodgkin-Huxley neurons, and the control performance is compared with existing control techniques. In addition, we demonstrate the robustness of the framework to system parameter variations as well as its generalizability to accommodate various physical restrictions, such as charge-balanced inputs.

## 2. Methods

### 2.1. Hodgkin-Huxley neuron model

We use Hodgkin-Huxley (HH) neuron model that describes the generation and propagation of action potentials in a squid giant axon based on the dynamic interplay between iconic conductances and electrical activity [32]. The dynamic equations describing the action potentials in a globally coupled heterogeneous neuron population are as follows:

$${\dot{V}}_{i}=\left[I_{b}+I(t)-{\bar{g}}_{Na,i}h\left(V_{i}-V_{Na,i}\right)m^{3}-{\bar{g}}_{K,i}\left(V_{i}-V_{k,i}\right)n^{4}-{\bar{g}}_{L,i}\left(V_{i}-V_{L,i}\right)\right]/c+\frac{\alpha }{N}\overset{N}{\underset{j=1}{\sum }}V_{j}-V_{i},$$

$${\dot{m}}_{i}=a_{m}\left(V_{i}\right)\left(1-m_{i}\right)-b_{m}\left(V_{i}\right)m_{i},$$

$${\dot{h}}_{i}=a_{h}\left(V_{i}\right)\left(1-h_{i}\right)-b_{h}\left(V_{i}\right)h_{i},$$

$${\dot{n}}_{i}=a_{n}\left(V_{i}\right)\left(1-n_{i}\right)-b_{n}\left(V_{i}\right)n_{i},$$

where $a_{m}\left(V_{i}\right)=0.1\left(V_{i}+40\right)/\left(1-\exp\left(-\left(V_{i}+40\right)/10\right)\right)$, $b_{m}\left(V_{i}\right)=4\;\exp\left(-\left(V_{i}+65\right)/18\right)$, $a_{h}\left(V_{i}\right)=0.07\;\exp\left(-\left(V_{i}+65\right)/20\right)$, $b_{h}\left(V_{i}\right)=1/1/\left(1+\exp\left(-\left(V_{i}+35\right)/10\right)\right)$, $a_{n}\left(V_{i}\right)=0.01\left(V_{i}+55\right)/\left(1-\exp\left(-\left(V_{i}+55\right)/10\right)\right)$, $b_{n}\left(V_{i}\right)=0.125\;\exp\left(-\left(V_{i}+65\right)/80\right)$ .

The axon membrane voltage of the neuron *i*, i=1,…,N is represented by the variable *V_i_*, while the ion gating variables are given by *m_i_*, *n_i_*, and *h_i_*. The baseline current and control input are given by *I_b_* and *I*, respectively, and *α* denotes the coupling strength between two neurons. The nominal parameters used in this paper are $V_{Na,i}=50\gamma_{i}$ mV, $V_{K,i}=-77\gamma_{i}$ mV, $V_{L,i}=-54.4\gamma_{i}$ mV, $I_{b}=10$ *μ*A/cm^**2**^, ${\bar{g}}_{Na,i}=120\gamma_{i}$ mS/ cm^2^, ${\bar{g}}_{K,i}=36\gamma_{i}$ mS/ cm^2^, ${\bar{g}}_{L,i}=0.3\gamma_{i}$ mS/ cm^2^ and *c* = 1*μ*F/cm^2^, where $\gamma_{i}$ is a varying parameter used to create heterogeneity in the neuron population.

### 2.2. Principles of data-driven control design

We consider the problem of designing a periodic input, *I*(*t*), that can desynchronize or synchronize an ensemble of coupled neurons in a given time *T*, where *T* is set to 10 × *T_mean_*, with *T*_*mean*_ denoting the mean natural period of the neurons. The periodic structure of the input is motivated by the findings that stimulation must be periodic in certain neurological treatments, such as deep brain stimulation [33]. Using Fourier representation, without loss of generality, we can express periodic input as

(1) $$I(t)=c_{0}+\overset{r}{\underset{m=1}{\sum }}c_{m}\cos(m\Omega t)+d_{m}\sin(m\Omega t),$$

where Ω is the mean natural frequency of the neurons and *r* is the number of Fourier harmonics in the input. By focusing on periodic input, the control design problem is equivalent to learning the input parameters $\beta ={\left[c_{0},c_{1},d_{1},\ldots ,c_{r},d_{r}\right]}^{\prime }$ that can produce the desired synchronization pattern. In the following, we describe our data-driven control scheme which includes (i) choosing an appropriate objective function ℒ depending upon the control objective and measurement availability, and (ii) learning the input coefficients β that minimize ℒ through systematic perturbations of the neuronal network.

We start by defining a function ℒ on the output measurements, individual or aggregated, so that minimization of ℒ achieves the desired synchronization structure in the neuronal ensemble. Given an input parameter vectors $\beta ={\left[c_{0},c_{1},d_{1},\ldots ,c_{r},d_{r}\right]}^{\prime }$, for small perturbations of the input coefficients, Δβ, the updated objective function due to the perturbations can be expressed as

$$\tilde{ℒ}=ℒ+\underset{≕𝒢}{\underset{︸}{\left[\begin{array}{c} \frac{\partial ℒ}{\partial c_{0}}\;\frac{\partial ℒ}{\partial c_{1}}\;\frac{\partial ℒ}{\partial d_{1}}\;\cdots \;\frac{\partial ℒ}{\partial c_{r}}\;\frac{\partial ℒ}{\partial d_{r}} \end{array}\right]}}\underset{≕\Delta \beta }{\underset{︸}{{\left[\begin{array}{c} \delta c_{0}\;\delta c_{1}\;\delta d_{1}\;\cdots \;\delta c_{r}\;\delta d_{r} \end{array}\right]}^{\prime }}}.$$

The importance of 𝒢 is that it encapsulates the local information of the system’s current dynamics and characterizes the effects of small input changes on the current trajectory. Our control idea is to leverage this expression in order to appropriately and gradually alter the control parameters, i.e., determine Δβ, to yield an improvement in the objective function. More specifically, to step-by-step steer the system closer to desired synchronization structure, in each iteration, we consider the following optimization problem

(2) $$\underset{\Delta \beta }{\text{minimize}}∥ℒ+𝒢\Delta \beta {∥}_{2}^{2}+\lambda ∥\Delta \beta {∥}_{2}^{2}$$

where λ≥0 is a regularization parameter to enforce a penalty on the magnitude of Δβ, ensuring that the input perturbations are small. We select λ in an adaptive manner such that $∥ℒ{∥}^{2}-∥\tilde{ℒ}{∥}^{2}\geq 2\sigma \left(∥ℒ{∥}^{2}-V_{\Delta \beta^{*},\lambda }\right)$ , where 1>σ>0 is a scaling factor and $V_{\Delta \beta^{*},\lambda }$ denotes the optimal objective value of equation (2). This condition is to ensure the updated control decreases the objective function (see Supplementary Materials for more details). In the absence of a mathematical model of the system, 𝒢 is estimated by perturbing each input coefficient and applying the associated perturbed input to compute the change in ℒ. After obtaining a (closed-form) solution to the quadratic program (2), i.e., $\Delta \beta^{*}=-{\left({𝒢}^{\prime }𝒢+\lambda I\right)}^{-1}{𝒢}^{\prime }ℒ$, we then update (or improve) the control parameters and repeat the process, which is outlined in the following algorithm.

**Algorithm 1:** Learning to control the synchronization structure of a neuron population

**Require:** Objective function ℒ, an initial (arbitrary) input coefficients β, a regularizer λ≥0, and α∈(0,1).
1: Apply the input corresponding to β, record the output trajectories, and compute the objective function ℒ.
2: Estimate 𝒢 by perturbing the input coefficients and observing the change in the objective function (estimated using the perturbed trajectories).
3: Solve for Δβ* of the optimization problem (2).
4. If $\|ℒ{\|}^{2}-\|\tilde{ℒ}{\|}^{2}\geq 2\sigma \left(\|ℒ{\|}^{2}-V_{\Delta \beta^{*},\lambda }\right)$, set λ=(1−α)λ. Else set λ=(1+α)λ and repeat step 3.
5: Update the input coefficients via $\beta =\beta +\Delta \beta^{*}$.
6: Repeat steps 1 – 5 until $\|ℒ{\|}^{2}\leq \epsilon_{\text{tolerance }}$ .

We prove that the proposed algorithm generates a sequence of parameters $\left\{\beta^{\left(k\right)}\right\}$, where *k* is the iteration number, such that $\left\{\left\|{ℒ}^{\left(k\right)}\right\|\right\}$ is a decreasing sequence that converges to 0. This is shown by leveraging the controllability properties of the population (see Supplementary Materials for proof). The subsequent subsections explain how to design an appropriate objective function ℒ for two scenarios: (i) when each neuron in the network can be measured, and (ii) when only an aggregated output measurement is available.

#### 2.2.1. When each neuron voltage can be measured individually

In scenarios where we can measure each individual neuron, the phase associated with the neuronal trajectory offers a practical and efficient way to measure the degree of synchrony in a neuron population. It is worth noting that, different from the phase-model control design, our approach only uses the concept of phase to characterize the level of synchrony of the neuron population and does not construct the phase model (from data) for the control design.

**Figure 2.**
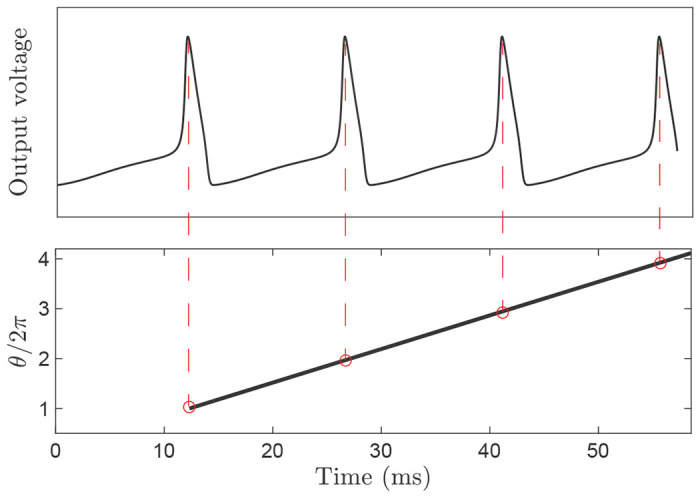
Phase estimation from the recorded voltage trajectory.

The phase of each neuron can be computed from its output voltage by considering the time gap between two consecutive neuron firings as one cycle; hence, the phase increase during the time interval equals 2π [22]. Therefore, by assigning a phase 2π*k* to time *t_k_*, time of *k*^*th*^ spike, the phase at time $t\left(t_{k}<t<t_{k+1}\right)$ can be determined using linear interpolation, i.e.,

(3) $$\theta (t)=2\pi k+2\pi \frac{t-t_{k}}{t_{k+1}-t_{k}},$$

where θ(t) is the phase at time *t* (Figure 2).

To evaluate the degree of network synchronization, we leverage the well-known Kuramoto order parameter defined as [34, 35]

(4) $$R^{2}={\left|\frac{1}{N}\sum_{i=1}^{N}e^{ı\theta_{i}}\right|}^{2}=\frac{1}{N^{2}}\left[{\left(\sum_{i=1}^{N}\cos\left(\theta_{i}\right)\right)}^{2}+{\left(\sum_{i=1}^{N}\sin\left(\theta_{i}\right)\right)}^{2}\right],$$

where *θ*_*i*_ is the estimated phase of neuron *i*, *i* = 1, … , *N*. The order parameter *R* indicates the amplitude of the mean-field behavior and takes values between *R* = 0, which corresponds to the incoherent state, and *R* = 1, which corresponds to the completely synchronized network state. Thus, a neuronal network can be desynchronized by designing an input *I*(*t*) of the form (1) that minimizes the order parameter, i.e., minimizes $ℒ=R^{2}$, at time *T*. Similarly, for the purpose of synchronization, the input of the form (1) is designed to maximize the Kuramoto order parameter, i.e., minimizes $ℒ=1-R^{2}$. Both tasks are achieved by applying Algorithm 1 with their corresponding objective functions ℒ.

#### 2.2.2. When only the aggregated voltage can be measured

The idea of leveraging the phases of individual neurons to evaluate the network synchrony falls short when the average output voltage of all neurons $V=N^{-1}\sum_{i=1}^{N}V_{i}$ is the only available quantity for measurement. In such scenarios, it becomes essential to assess the network synchrony from the observed mean voltage. We do this by leveraging a physical observation, that is, synchronization results in enhanced oscillations of the mean-field, while desynchronization manifests itself via reduced mean-field oscillations [11, 22, 36]. Neurons can therefore be synchronized (desynchronized) by amplifying (inhibiting) the mean field oscillations, which is achieved by increasing (decreasing) the variance of the mean field. By utilizing this insight, for the desynchronization objective, we inhibit the mean voltage oscillations by minimizing the objective function

(5) $$ℒ\left(V^{\left(1\right)},\ldots ,V^{\left(M\right)}\right)=\mathrm{Var}(V)=\frac{1}{M}\sum_{k=1}^{M}{\left(V^{\left(k\right)}-\bar{V}\right)}^{2}$$

where Var(V), $\bar{V}$ are the numerically estimated variance and mean using the *M* mean filed samples, $V^{\left(1\right)},\ldots ,V^{\left(M\right)}$ (*M* samples are collected during the last measurement cycle). Similarly, to synchronize the population we amplify the mean voltage variance by minimizing ℒ=K−Var(V), where *K* is a large enough positive number to ensure L≥0 .

## 3. Results

### 3.1. Regulating synchrony through individual voltage measurements

**Figure 3.**
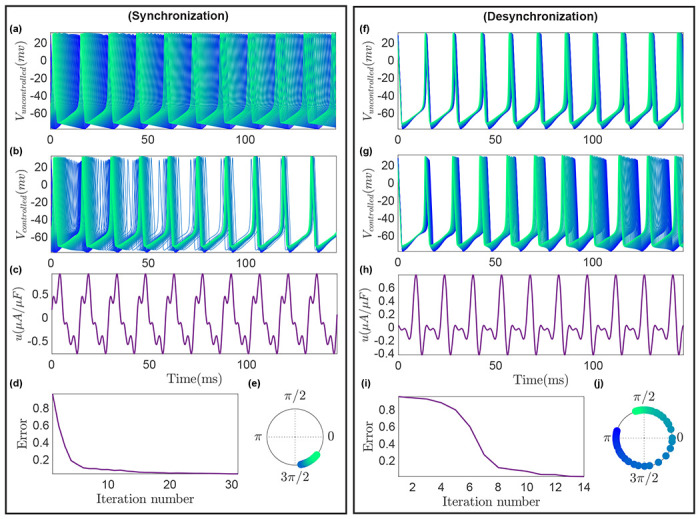
Synchronization (left) and desynchronization (right) of a population of 50 Hodgkin-Huxley coupled nonidentical neurons. Voltage measurements of individual neurons are available and thus utilized to estimate the neuron’s phases for the control design purpose. **(a)**, **(f)** Voltage traces for the individual neurons when no control is applied. **(b), (g)** Voltage traces for the individual neurons when the control is applied. **(c), (h)** Final control input obtained after learning. **(d), (i)** Convergence behavior of the objective function. **(e), (j)** Final phases of the neurons on a unit circle.

We consider the objective of desynchronizing a population of globally coupled 50 HH heterogeneous neurons (γ∈[0.95,1.05]) when each individual neuron can be measured. In the absence of external input, this population remains synchronized due to internal coupling $\alpha =5\times 10^{-2}$ (Figure 3(f)). To break the simultaneous neuron firing, we apply the proposed data-driven control technique by initializing the input to be zero, i.e., β=0. The input coefficients are then slightly perturbed and 𝒢 is estimated from changes in the objective function ℒ due to small input perturbations, which is used to update the input (equation (2)). This process is repeated 14 times till ‖ℒ‖<0.01, and the resulting neuron voltages are shown in Figure 3(g). Panels (h), (i), and (j) of Figure 3 display the designed control, ‖ℒ‖ variation with each iteration, and the estimated final neuron phases. Note that our approach does not require any data-intensive task, such as training an artificial neural network, for control design and only takes 14 iterations to achieve desynchronization, significantly fewer than other online learning approaches such as Reinforcement Learning [29, 30].

Now, we consider the synchronization of 50 coupled HH neurons where the population is in a desynchronized state in the absence of external input (Figure 3(a)) due to heterogeneity (γ∈[0.95,1.05]) and weak coupling $\alpha =2.5\times 10^{-3}$. Similar to the previous case, we initialize the input parameters to zero and then employ Algorithm 1 till ‖ℒ‖<0.01. After 30 iterations, the neuron population is successfully synchronized. The controlled trajectories, designed input, ‖ℒ‖ in each iteration, and the estimated neuron phases in the final cycle are shown in Figures 3(b)–(e). It is worth noting that to archive (de)synchronization, some existing data-driven neuronal control methods in fact require a rather complete set of pre-training data with a uniform distribution [25–27]. In practical applications, such complete data sets are often not readily available and must be carefully collected during experiments. Our approach, on the other hand, does not rely on pre-training data and only utilizes the data in the neighborhood of the system’s current trajectory to facilitate control design.

In addition, we consider (de)synchronizing population of identical HH neurons. Our algorithm takes 31 (22) iterations to synchronize (desynchronize) the population (see Supplementary Materials Figure 2). These results illustrate that the proposed data-driven technique is effective in both enhancing and suppressing the synchronization of neuron populations when individual neuronal measurements are available.

### 3.2. Learning control from a population-level measurement

**Figure 4.**
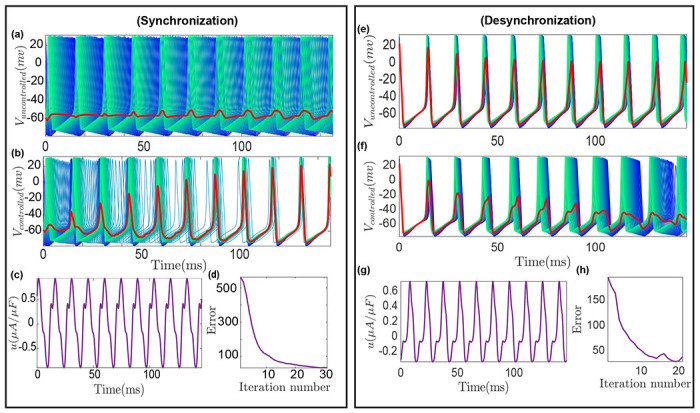
Synchronization (left) and desynchronization (right) of a network of 50 Hodgkin-Huxley neurons using noisy aggregated measurements. The neuron population includes weakly coupled and nonidentical units in which only the noisy mean firing voltage can be observed. (a), (e) Voltage traces for the individual neurons and the aggregated voltage measurement (red line) when no control input is applied. (b), (f) Voltage traces for the individual neurons and the aggregated voltage measurement (red line) when the control is applied. (c), (g) Final control input obtained after learning. (d), (h) Convergence behavior of the control objective function.

We consider the (de)synchronization of 50 coupled HH neurons where the noisy mean firing voltage of the population is the only available measurement. This is achieved by designing a control input to suppress (for desynchronization) or enhance (for synchronization) mean field oscillations using Algorithm 1. The results are displayed in Figure 4, where desynchronization, similarly synchronization, is achieved when the mean voltage (red line) oscillations are reduced, similarly amplified (Figures 4(b) and (f)). Note in Figure 4(f), the lower the mean field oscillations, the more desynchronized the population is. Additionally, similar to the case of individual neuron measurement, our algorithm requires 14 (31) iterations to achieve desynchronization (synchronization) (Figures 4(d) and (i)) even with a single noisy population-level output measurement.

Now, we compare the control performance, input power and error in the control objective, of the individual measurement case to the aggregated measurement case (Table I). The input power is estimated as $\frac{1}{T_{I}}\int_{T_{I}}I^{2}(t)dt=c_{0}^{2}+\frac{1}{2}\sum_{m=1}^{2}c_{m}^{2}+d_{m}^{2}\left(T_{I}=2\pi /\Omega \right)$ using Parseval’s theorem [37], and the error in control objective is taken as 1 – *R*^2^ for synchronization and *R*^2^ for desynchronization. Note that we estimate the order parameter, *R*^2^, from the individual voltage trajectories in the aggregated case only for comparison purposes. We observe that the control performance of our algorithm with aggregated measurement is similar to the individual measurement case for the desynchronization objective (Table I). However, we observe a small increase in the input power for the synchronization case.

### 3.3. Online Dynamic Adaption to System Variation

**Figure 5.**
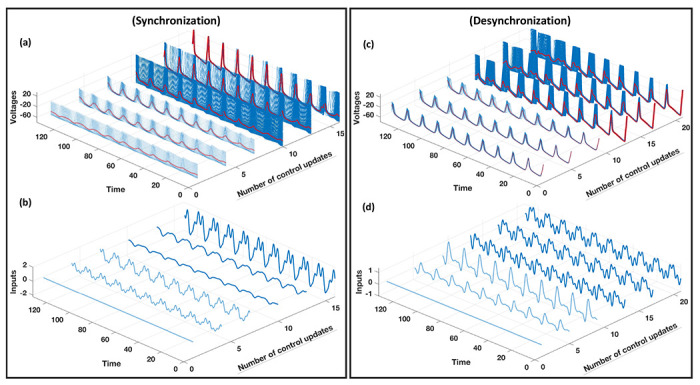
Synchronization (left) and desynchronization (right) of a coupled network of 50 nonidentical Hodgkin-Huxley neurons using noisy aggregated measurements. The neuronal population varies its parameters during the control process where the light blue (dark blue) color denotes the system before (after) the change. (a), (c) Evolution of individual neurons’ voltages and of the aggregated voltage measurement (red line) during the control process. (b), (d) Evolution of the corresponding inputs that are applied to the system during the control process.

Unquestionably, system variation is a distinct characteristic of biological and physical rhythmic systems, such as fluctuations of the interspike interval in real neuron activity [38] and frequency variability in electrochemical oscillator networks [39]. Therefore, it is essential to develop control techniques that can accommodate variations in the underlying system. To illustrate this aspect of our method, we consider a HH neuron population with the model parameter *γ*_*i*_ changes from $\gamma_{i}^{\left(1\right)}\in [0.75,0.85]$ to $\gamma_{i}^{\left(2\right)}\in [0.95,1.05]$. This variation occurs during the learning process and alters the spiking behavior of the neurons, as displayed in Figure 5(a) and (c), where light blue (dark blue) spikes correspond to $\gamma_{i}^{\left(1\right)}\gamma_{i}^{\left(2\right)}$). Our iterative control technique quickly adapts to these changes and achieves the desired synchronization structures. This adaptation of control is evident from Figure 5(b) and (d) (light blue to dark blue). Even with such system variation, the total number of control updates remains low, with 16 (21) times for synchronization (desynchronization).

It is worth noting that controlling either synchronization or desynchronization of the severely underactuated HH population through a single control and a single aggregated noisy measurement is a highly non-trivial task even when the system model is available [40–43]. To the best of our knowledge, no other data-driven control work has addressed this issue. In this paper, we achieve this control task purely by means of online data from a small number of trials while being able to handle changes in the system with minimal degradation in control performance.

### 3.4. Robustness to coupling strength variation

In a population of coupled HH neurons, synchronization is observed when the intrinsic coupling strength *α* is increased [44–46]. This means that as coupling strength increases, less external forcing is necessary for synchronization, while strong input is required for desynchronization to dominate the system dynamics. A similar behavior is observed when we construct control inputs to synchronize or desynchronize a neural population for various coupling strengths (Table II). Specifically, we observe that the control input power required for synchronization decreases as the *α* increases, whereas the opposite trend is found for desynchronization control. These results have two implications: (i) our control method performs consistently over a wide variety of coupling strengths, and (ii) the control technique leverages the system dynamics to find a relatively minimal control rather than merely finding a feasible control. Note that, for the purpose of comparison, the error tolerance ϵ_tolerance_ is kept identical across all coupling strengths.

### 3.5. Comparison with model-based methods

**Figure 6.**
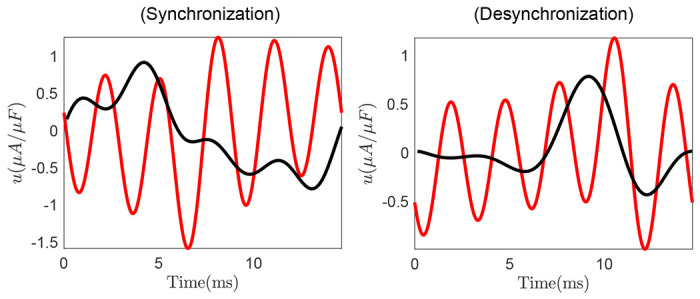
Control inputs generated using our model-free method (black) and the model-based technique (red) to synchronize (left panel) or desynchronize (right panel) a neural population.

Here, we show that our data-driven control method achieves similar performance as a model-based control design techniques, such as [15]. The technique in [15] utilizes the phase models to construct periodic, open-loop control inputs that synchronize an ensemble of heterogeneous oscillators. We keep the input frequency similar for both methods and compare the input power and the accuracy of the final synchronization pattern. We consider a network of 50 coupled non-identical HH neurons and design control inputs to desynchronize the network from a synchronized state (and, similarly, to synchronize from a desynchronized initial state). The comparison results are displayed in Table III with the corresponding control inputs shown in Figure 6. This comparison illustrates that the proposed model-free technique achieves better control performance, both in terms of the control objective ℒ and input power, than the model-based technique. For the synchronization objective, our approach delivers comparable synchronization performance with 60% less power, whereas for the desynchronize objective, we obtain an improvement of 52% in the control objective with a three-fold reduction in input power. This improvement in control performance can be explained by the fact that the model-based approach imposes certain assumptions, such as network topology and coupling structure, on the system dynamics for the control design, whereas our model-free strategy learns the control input by directly interacting with the system without any assumptions.

## 4. Discussion

In this paper, we present a data-driven framework to effectively regulate the synchrony in a network of coupled heterogeneous neurons. We apply the proposed framework to the HH neuron population and show that the desired controls can be determined from noisy aggregated measurements. We provide a theoretical analysis of the convergence of our algorithm. Further, through the means of numerical simulations, we show that our data-driven controls provide better performance than the classic phase model-based approaches.

Our control technique differs from the existing data-driven techniques for complex networks [25–27, 29–31] in two important aspects. Firstly, we do not extract information from a rich pre-training data set, as in [25–27], and directly learn the appropriate control online from scratch. Online interactions with the system enable the control synthesis to effectively adapt to system variation. Secondly, unlike Reinforcement Learning [25–27], we do not learn from a limited reward signal, which without a hand-crafted function requires many trials with a large amount of data. Instead, we utilize the rich local dynamics of the system along its current trajectory to step-by-step regulate the system toward the desired synchrony.

The suggested control method is both theoretically sound and practically applicable, as it merely requires the (population-level) voltage measurement to learn the desired control without relying on any assumptions on the system dynamics. The online learning aspect of our algorithm enables us to effectively regulate systems with time-varying parameters. This problem of controlling time-varying systems, which is rather challenging in a model-based setting, finds a simpler solution in a data-driven framework due to the continual nature of online system interactions. In addition, our algorithm offers the generalizability to incorporate experimental constraints, such as charge-balanced (CB) inputs, which is a necessary requirement for deep-brain stimulation [16, 47]. This CB constraint can be expressed as $\int_{0}^{T}I(t)dt=0$, where *T* is the duration of the stimulation [19], and can readily be implemented in our framework by setting *c*_0_ = 0.

Some limitations of our work should also be addressed and acknowledged. First, the evaluation of the gradient from the perturbed trajectory might suffer from noise. This can be mitigated by ensuring that the perturbation is larger than the noise. Second, our algorithm requires resetting the system to evaluate the effect of perturbation, which can be challenging in certain applications. However, the number of resettings required by our algorithm is low, which can be done by applying a resetting pulse in an experimental setup. Nonetheless, it would be ideal to have a data-driven strategy that does not require the reset of experiments, since this would enhance the relevance and application of our data-driven control framework and lead to practical control methods for complex networks.

## Figures and Tables

**Table I.** A comparison of control performance between the individual and aggregated measurement cases.

	Synchronization		Desynchronization	
	Individual	Aggregated	Individual	Aggregated
Error	0.02	0.06	0.008	0.02
Input Power	0.24	0.33	0.1	0.11

**Table II.** Effect of coupling strength on input power.

Coupling strength	Synchronization	Desynchronization
10^−5^	0.2738	0.033
10^−4^	0.2243	0.036
5 × 10^−4^	0.1957	0.06
8 × 10^−4^	0.0145	0.08
10^−3^	0.0046	0.1

**Table III.** Control performance with the proposed approach and the reference model-based technique proposed in [15].

	Synchronization		Desynchronization	
	Proposed	Reference	Proposed	Reference
Error	0.02	0.01	**0.008**	0.52
Input Power	**0.24**	0.59	**0.1**	0.3

## Data Availability

All data generated or analyzed during the study are included in this article (and its Supplementary Materials). The simulation codes are further available upon request.
